# Microbiome characteristics associated with lymph node metastasis in laryngeal squamous cell carcinoma

**DOI:** 10.1038/s41598-025-16431-5

**Published:** 2025-08-24

**Authors:** Fangxu Yan, Shibo Chen, Xin Xia, Yue Fan, Shuting Yu, Xiao Zhang, Xingming Chen

**Affiliations:** 1 Department of Otolaryngology-Head and Neck Surgery, Peking Union Medical College Hospital, Peking Union Medical College and Chinese Academy of Medical Sciences, No. 1, Shuaifuyuan, Wangfujing, Beijing, 100730 China; 2 Institute of Artificial Intelligence, Beihang University, Beijing, 100191 China; 3 School of Mathematical Sciences, Beihang University , Beijing, 100191 China; 4 Key Laboratory of Mathematics, Informatics and Behavioral Semantics (LMIB), Ministry of Education, Beijing, 100191 China; 5 Zhongguancun Laboratory, Beijing, 100094 China; 6 Hangzhou International Innovation Institute of Beihang University, Hangzhou, 311115 China

**Keywords:** Microbiome, Laryngeal squamous cell carcinoma, Biomarker, 16S rRNA, Lymph node metastasis, Head and neck cancer, Microbiome

## Abstract

Lymph node (LN) metastasis is a key prognostic factor in laryngeal squamous cell carcinoma (LSCC). Emerging evidence implicates the role of the microbiome in cancer progression. This study aimed to investigate the microbial features associated with lymph node metastasis in LSCC and their potential for patient stratification. Using 16 S rRNA gene sequencing, we characterized the microbiome of tumor tissues, adjacent normal tissues, lymph nodes, and oral rinses from 108 LSCC patients, including 36 with (LN+) and 72 without (LN-) cervical LN metastasis. Microbial functional potential was predicted using PICRUSt2. Based on repeated stratified 3 cross-validation, random forest models were used to identify metastasis-associated genera. Significant microbial differences were observed between LN + and LN- tumor tissues, with Ralstonia enriched in LN + tumors and Fusobacterium more abundant in LN- cases. All genera detected in lymph nodes were also found in tumor tissues. Functional predictions revealed enrichment of lipid biosynthesis, energy metabolism, and cell wall synthesis pathways in LN + patients, particularly in tumor and oral rinse samples, with low intra-group variability. Classifiers based on tumor, lymph node, and oral microbiota demonstrated the ability to distinguish LN + from LN- patients. The lymph node-derived classifier achieved an accuracy of 84.31% (95% confidence interval [CI]: 81.76% − 86.85%), followed by the tumor-based model (AUC = 84.11%, 95% CI: 81.75% − 86.46%) and oral rinse classifier (AUC = 79.88%, 95% CI: 77.09% − 83.11%). A tumor-specific 17 genera panel showed a discriminative efficacy of 84.11% (95% CI: 81.75% − 86.46%) in tumor tissues. These findings suggest that microbiome alterations may be associated with lymph node metastasis in LSCC. In addition, the oral microbiome showed potential as a non-invasive tool for occult lymph node metastasis detection. However, these results are preliminary and require validation in larger, independent cohorts.

**Supplementary Information:**

The online version contains supplementary material available at 10.1038/s41598-025-16431-5.

## Introduction

Laryngeal cancer is a common malignancy that arises in the head and neck region, with more than 95% of which being squamous cell carcinoma^1^. In 2020, there were 184,615 newly diagnosed cases of laryngeal cancer worldwide, resulting in 99,840 deaths^2^. Despite recent advancements in diagnosis and treatment, approximately 60% of laryngeal squamous cell carcinoma (LSCC) patients present with an advanced stage at diagnosis, and the prognosis of these patients has not significantly improved despite the advent of multi-disciplinary treatment (MDT)^3^. Cervical lymph node (LN) metastasis is one of the leading adverse prognostic factors of LSCC^1^. Once lymph node metastasis occurs, the chance of radical treatment will decrease by 50%, and overall survival is significantly reduced^4^. However, the mechanisms underlying lymph node metastasis in LSCC remain incompletely understood, hindering the development of targeted therapeutic strategies. Furthermore, the current clinical evaluation mainly relies on pre-treatment imaging and ultrasound, which are insufficient for effectively identifying occult lymph node metastasis. There is an urgent need for reliable biomarkers to predict and detect lymph node metastasis at an early stage.

The human microbiome is deemed to be a “second genome of the host”^5,6^. The microbiome has been established as a crucial component of the tumor microenvironment, contributing to tumorigenesis not only by inducing DNA damage and activating oncogenic signaling pathways but also by modulating local inflammation and immune responses^7–9^. For example, *Fusobacterium nucleatum* has been shown to enhance the migration and adhesion of oropharyngeal squamous cell carcinoma (OSCC) cells while suppressing the antitumor activity of immune cells^10–12^. Lymph nodes, serving as central hubs for immune surveillance, represent primary metastatic sites in cancer progression. Michikawa et al. reported a strong association between primary tumor immune infiltration and lymph node metastasis in oral cancer, with non-metastatic tumors demonstrating reduced immune infiltration compared to metastatic tumors lacking extranodal extension^13^. Collectively, these findings indicate that microbiota-mediated alterations in tumor cell biology and immune microenvironment remodeling may contribute to lymph node metastasis.

Previous studies have established associations between the microbiome and LSCC^14–16^. Our previous work also demonstrated significant differences in the oral and tumor microbial profiles between LSCC patients and non-cancer controls, highlighting the potential of the oral microbiome as a diagnostic biomarker for LSCC^17^. However, the relationship between microbiome and lymph node metastasis remains poorly understood. Current studies on the role of microbiome in lymph node metastasis of head and neck cancers are limited, and have primarily focused on oral or oropharyngeal cancers^18,19^with little attention to LSCC. In addition, most existing studies have analyzed either tissue (tumor, lymph node, etc.) or oral (oral rinses, swabs, saliva, etc.) samples in isolation, to some extent, restricting cross-site comparability and biological interpretability. In this study, we aimed to characterize the microbial features of tumor tissues, adjacent normal tissues, lymph node tissues, and oral rinses in LSCC patients with (LN+) and without (LN-) lymph node metastasis. Furthermore, we sought to develop a microbiome-based classification model to distinguish LN + and LN- patients, providing novel insights for the clinical management of LSCC.

## Materials and methods

### Sample collection

A total of 108 patients newly diagnosed with LSCC at Peking Union Medical College Hospital (PUMCH) between 2014 and 2023 were recruited in this study. All patients underwent radical resection of laryngeal cancer, and some patients received cervical lymph node dissection. The participants were divided into two groups according to the presence or absence of cervical lymph node metastasis with pathological confirmation, including 36 LSCC patients with lymph node metastasis and 72 without lymph node metastasis. Patients who had taken any antibiotics in the past 30 days or with a history of other malignancies, chemotherapy, or radiotherapy were excluded. The collected data of patients included demographic information (i.e., age, sex, smoking status, and alcohol consumption) and clinical characteristics (i.e., primary tumor subsite, histologic grade, tumor size, clinical and pathological TNM stage, prognostic stage, lymph node dissection findings, and extranodal extension status). Patients who had smoked more than 100 cigarettes in their lifetime were classified as “Smokers”, while those who had smoked fewer were considered “never smokers”. “Drinkers” were defined as individuals who consumed alcohol at least once a week for a year or more, whereas those who drank less frequently were classified as “Non-drinkers”. The present study was conducted according to the Helsinki Declaration and obtained approval from the Ethics Committee of Peking Union Medical College Hospital (approval ID number: ZS-3148). All patients signed informed consent before participation.

We aimed to collect oral rinses, tumor tissues, adjacent normal tissues, and lymph node tissues from LSCC patients. The oral rinse samples were obtained before surgery. The patients were required to avoid eating, drinking, and brushing for at least 2 h before sample collection. Each patient then gargled fully with 10 ml of sterile saline for 30 s and expelled the rinse into a sterile collection tube. Intraoperative tissue specimens were immediately harvested post-resection under strict aseptic protocols. Tumor tissues were excised from the center region of neoplastic masses, while adjacent normal tissues were obtained from areas at least 1 cm away from the margin of the tumor^15^. Harvested lymph nodes were halved, one into sterile tubes for microbial profiling and the other for histopathological examination to confirm metastatic status. All samples were immediately transferred to long-term storage at – 80 °C.

Due to clinical practice limitations, we were unable to match all the samples for each patient. In practice, samples were collected opportunistically based on surgical accessibility and patient condition. A total of 80 tumor tissues, 25 adjacent normal tissues, 39 lymph node tissues, and 53 oral rinses were obtained from 108 LSCC patients. Among these, a subset of patients provided two or more sample types. 30 patients provided matched tumor and lymph node samples, and 29 patients had matched tumor and oral rinse samples. The full distribution and matching combinations are visualized in the sample collection flowchart (Supplementary Figure S1).

### 16S rRNA sequencing analysis

Total genomic DNA was extracted from the samples using the QIAampFast DNA Stool Mini Kit (Qiagen, Hilden, Germany). The V3-V4 region of the 16S rRNA gene was amplified by PCR using the primers 341 F and 806R. Amplified PCR products were then purified with the GeneJET Gel Extraction Kit (Thermo Fisher Scientific). Sequencing libraries were constructed using the NEB Next^®^ Ultra™ DNA Library Prep Kit (New England Biolabs) with index codes added for sample identification. Finally, qualified libraries were sequenced on the Illumina NovaSeq platform, generating 250 bp paired-end reads.

### Bioinformatics and statistical analysis

We compared the baseline characteristics of LSCC patients with and without lymph node metastasis using R software (version 4.4.2). Categorical variables were analyzed using the chi-square test, while continuous variables were assessed using either the Student’s t-test or the Wilcoxon rank-sum test, as appropriate. All tests were two-sided, with *P* < 0.05 considered statistically significant.

Bioinformatics analysis was performed on the 16 S rRNA sequencing data. Paired-end reads were merged using FLASH^20^ and low-quality reads were filtered using QIIME (version 1.8)^21^. Chimeric sequences were removed using the USEARCH algorithm (version 6.1)^22^ and high-quality sequences were clustered into operational taxonomic units (OTUs) at a 97% similarity threshold in QIIME. The corresponding OTU table has been provided as Supplementary Table S1. Taxonomic annotation of representative OTU sequences was conducted based on the Greengenes database (version 13.8)^23^. To characterize the microbial community, α-diversity was assessed using the Simpson, Shannon, Chao1, and Observed OTUs indices, with intergroup comparisons performed using the Wilcoxon rank-sum test. Principal coordinates analysis (PCoA) based on the Bray-Curtis distance matrix was used to visualize β-diversity, and microbial community structure differences between groups were evaluated using permutational multivariate analysis of variance (PERMANOVA). Linear discriminant analysis effect size (LEfSe) was employed to identify differentially abundant genera between the LN + and LN- groups, with a Kruskal-Wallis significance threshold set as α = 0.05 and a logarithmic linear discriminant analysis (LDA) score threshold of 2.0. Venn diagrams were generated to visualize shared taxa across different sample types, and heatmaps were constructed to display genus-level relative abundance profiles.

Microbial functional profiles were predicted using phylogenetic investigation of communities by reconstruction of unobserved states (PICRUSt2, version 2.6.2), based on 16 S rRNA gene sequencing data from tumor tissues, adjacent normal tissue, lymph node tissues, and oral rinses. Functional pathway annotation was performed using the MetaCyc database, and pathway abundances were inferred using the MinPath algorithm. Differentially enriched metabolic pathways were further identified using LEfSe analysis, with an LDA score cutoff of 1.5 and a significance threshold of α = 0.05.

Using the randomForest package in R, we constructed random forest (RF) classifiers based on microbial profiles from tumor tissues, lymph node tissues, and oral rinses to distinguish LN + from LN- LSCC patients. The mean decrease in accuracy (MDA) method was used to assess the importance of each genus, and the top 25 genera ranked by MDA were selected as key features. The model was then validated through 20 repetitions of stratified three-fold cross-validation. The discriminative performance of the classifiers was evaluated using receiver operating characteristic (ROC) curves and the area under the curve (AUC), with higher AUC values indicating better classification performance. PERMANOVA based on Bray-Curtis distance matrix was performed using the top 25 genera to evaluate the biological relevance of features selected by the model. Analyses were conducted separately for tumor, lymph node, and oral rinse samples to compare community structure between LN + and LN- groups.

## Result

### Patient characteristics

This study included 36 LSCC patients with lymph node metastasis and 72 without lymph node metastasis. The demographic characteristics of the study cohort are presented in Table 1. There were no significant differences between the two groups in terms of age, sex, smoking status, or alcohol consumption (*P* > 0.05). The clinical data of the patients are summarized in Supplementary Table S2.

**Table 1:** Demographic and clinical characteristics of participants.

Characteristics	All patients (*n* = 108)	Patients with metastasis (*n* = 36)	Patients without metastasis (*n* = 72)	*P* value
Age, mean (SD), years	60.17 ± 8.89	61.94 ± 7.77	59.28 ± 9.33	0.143
Sex, n (%)				0.398
Male	102(94.4)	33(91.7)	69(95.8)	
Female	6(5.6)	3(8.3)	3(4.2)	
Smoking status, n (%)				0.056
Smokers	95(88.0)	35(97.2)	60(83.3)	
Non-smokers	13(12.0)	1(2.8)	12(16.7)	
Alcohol consumption, n (%)				0.123
Drinkers	67(62.0)	26(72.2)	41(56.9)	
Non-drinkers	41(38.0)	10(27.8)	31(43.1)	

*P* < 0.05 is considered statistically significant.

### Comparison of microbial diversity between LSCC patients with and without lymph node metastasis

**Fig. 1:**
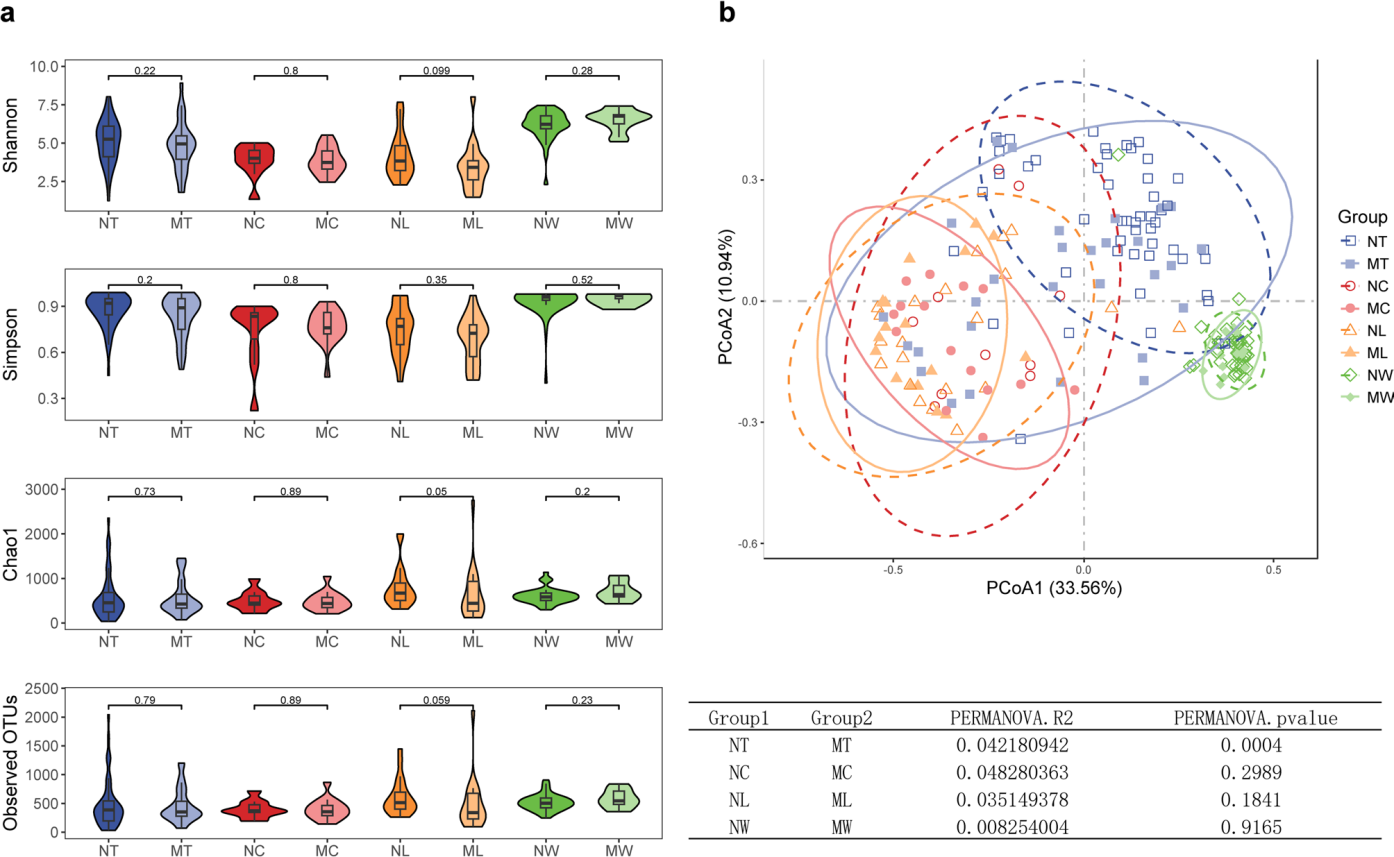
Microbial diversity in tumor tissues, adjacent normal tissues, lymph node (LN) tissues, and oral rinses samples of LSCC patients with (LN+) and without (LN-) lymph node metastasis. **(a)** Comparison of α-diversity between groups. *P* values were calculated using the Wilcoxon rank-sum test. **(b)** Principal coordinate analysis (PCoA) plot. *P* values were derived from permutational multivariate analysis of variance (PERMANOVA). *P* < 0.05 was considered statistically significant. NT, tumor tissues from LN- patients; MT, tumor tissues from LN + patients; NC, adjacent normal tissues from LN- patients; MC, adjacent normal tissues from LN + patients; NL, non-metastatic lymph node tissues; ML, metastatic lymph node tissues; NW, oral rinses from LN- patients; MW, oral rinses from LN + patients.

We assessed the microbial diversity in the LN + and LN- groups of LSCC across different sample types. For α-diversity, compared to LN- patients, LN + patients exhibited lower microbial richness in tumor tissues, adjacent normal tissues, and lymph node tissues, whereas the inverse trend was observed in oral rinse samples, as measured by Shannon, Simpson, Chao1, and Observed OTUs indices. However, none of these differences reached statistical significance (Fig. 1a). For β-diversity, PCoA based on Bray-Curtis distances at the genus level revealed distinct clustering between LN + and LN- groups in tumor tissues (*P* = 0.0004, PERMANOVA), whereas adjacent normal tissues and lymph node tissues showed no significant compositional differences (Fig. 1b). Additionally, compared to tissue samples, microbial taxa in oral rinse samples from both groups exhibited a closer clustering pattern.

### Microbial structural discrepancy in lymph node metastasis and non-metastasis groups of LSCC

**Fig. 2:**
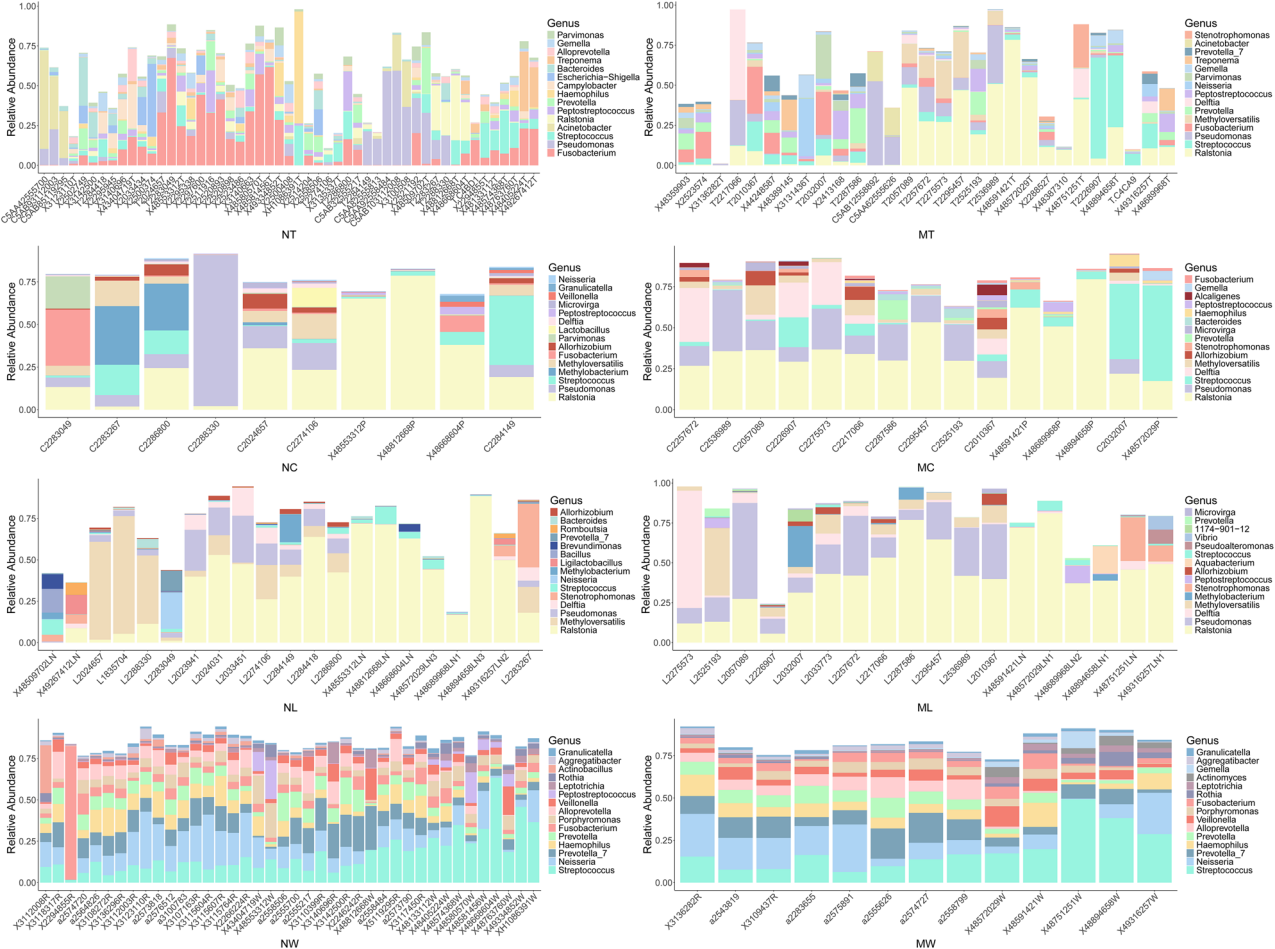
The relative abundance of the top 15 genera in tumor tissue, adjacent normal tissue, lymph node (LN) tissue, and oral rinse samples. NT, tumor tissues from LN- patients; MT, tumor tissues from LN + patients; NC, adjacent normal tissues from LN- patients; MC, adjacent normal tissues from LN + patients; NL, non-metastatic lymph node tissues; ML, metastatic lymph node tissues; NW, oral rinses from LN- patients; MW, oral rinses from LN + patients.

We compared the relative abundances of the top 15 genera across different sample types (tumor tissues, adjacent normal tissues, lymph node tissues, and oral rinses) in LSCC patients with and without lymph node metastasis (Fig. 2). In tumor tissues, significant differences in microbial composition were observed between the LN + and LN- groups. Specifically, *Ralstonia* accounted for a substantially higher proportion in LN + tumor tissues compared to LN- tumors (0.172 ± 0.212 (mean ± SD) vs. 0.034 ± 0.079, LN + vs. LN- tumor tissues), whereas *Fusobacterium* was more prevalent in LN- tumors (0.044 ± 0.077 vs. 0.138 ± 0.169). In contrast, adjacent normal tissues and lymph node tissues exhibited similar microbial profiles between groups, with *Ralstonia* remaining the most abundant genus in both sample types, regardless of metastatic statu. For oral rinse samples, the overall microbial composition was more similar between LN + and LN- patients. *Streptococcus*,* Neisseria*, and *Prevotella_7* were consistently the top three abundant genera in both groups. Full statistical data, including percentage distributions and *P* values for all genera across comparative groups (Supplementary Table S3).

**Fig. 3:**
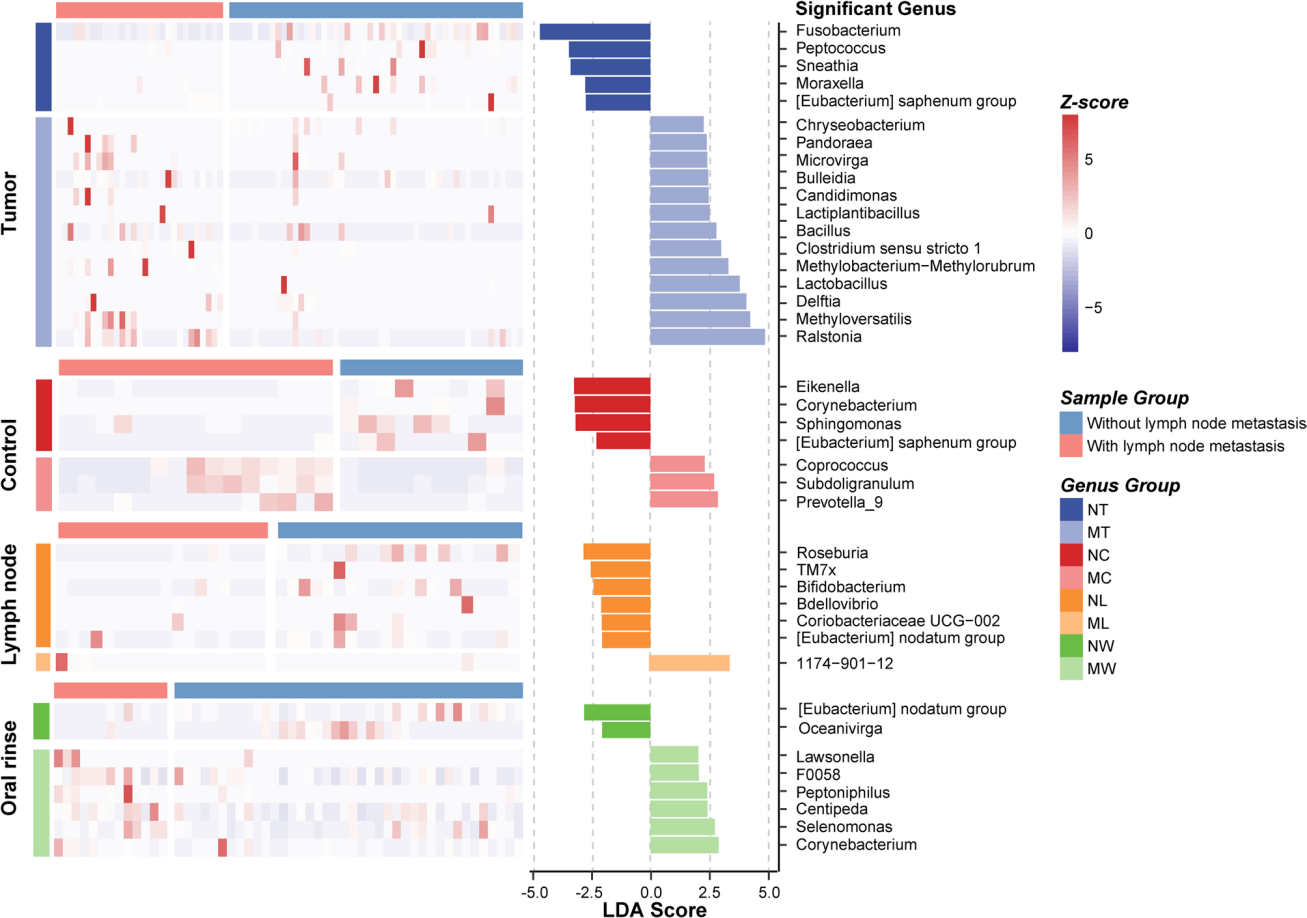
Differential microbial composition between LN + and LN- groups in LSCC patients across tumor tissue, adjacent normal tissue, lymph node tissue, and oral rinse samples. A linear discriminant analysis (LDA) score threshold of |LDA Score| ≥ 2 was used to identify genera with significant differences between LN + and LN − LSCC patients across different sample types. The bar plot displays these differentially abundant genera, while the heatmap illustrates their relative abundance. NT, tumor tissues from LN- patients; MT, tumor tissues from LN + patients; NC, adjacent normal tissues from LN- patients; MC, adjacent normal tissues from LN + patients; NL, non-metastatic lymph node tissues; ML, metastatic lymph node tissues; NW, oral rinses from LN- patients; MW, oral rinses from LN + patients.

LEfSe analysis identified differentially abundant genera between LN + and LN- groups across various sample types, with heatmaps illustrating relative abundance patterns (Fig. 3). In tumor tissues, the relative abundance of *Ralstonia*, *Methyloversatilis*, *Delftia*, *Lactobacillus*, and *Methylobacterium-Methylorubrum* was significantly higher in the LN + group than in the LN- group. Conversely, *Fusobacterium*, *Peptococcus*, *Sneathia*, *Moraxella*, and *[Eubacterium] saphenum group* were significantly less abundant in LN + tumor tissues. Metastatic and non-metastatic lymph nodes also exhibited distinct microbial profiles. *Bdellovibrio*, *1174-901-12*, and *Ramlibacter* were significantly enriched in metastatic lymph nodes, whereas *Roseburia*, *TM7x*, *Coriobacteriaceae UCG-002*, *[Eubacterium] nodatum group*, *Bifidobacterium*, and *Monoglobus* were more abundant in non-metastatic lymph nodes. For oral rinse samples, several genera displayed significant differences between LN + and LN- patients. In LN + patients, the relative abundance of *Corynebacterium*, *Selenomonas*, *Centipeda*, *Peptoniphilus*, *F0058*, and *Lawsonella* was elevated, while *[Eubacterium] nodatum group* and *Oceanivirga* were reduced.

**Fig. 4:**
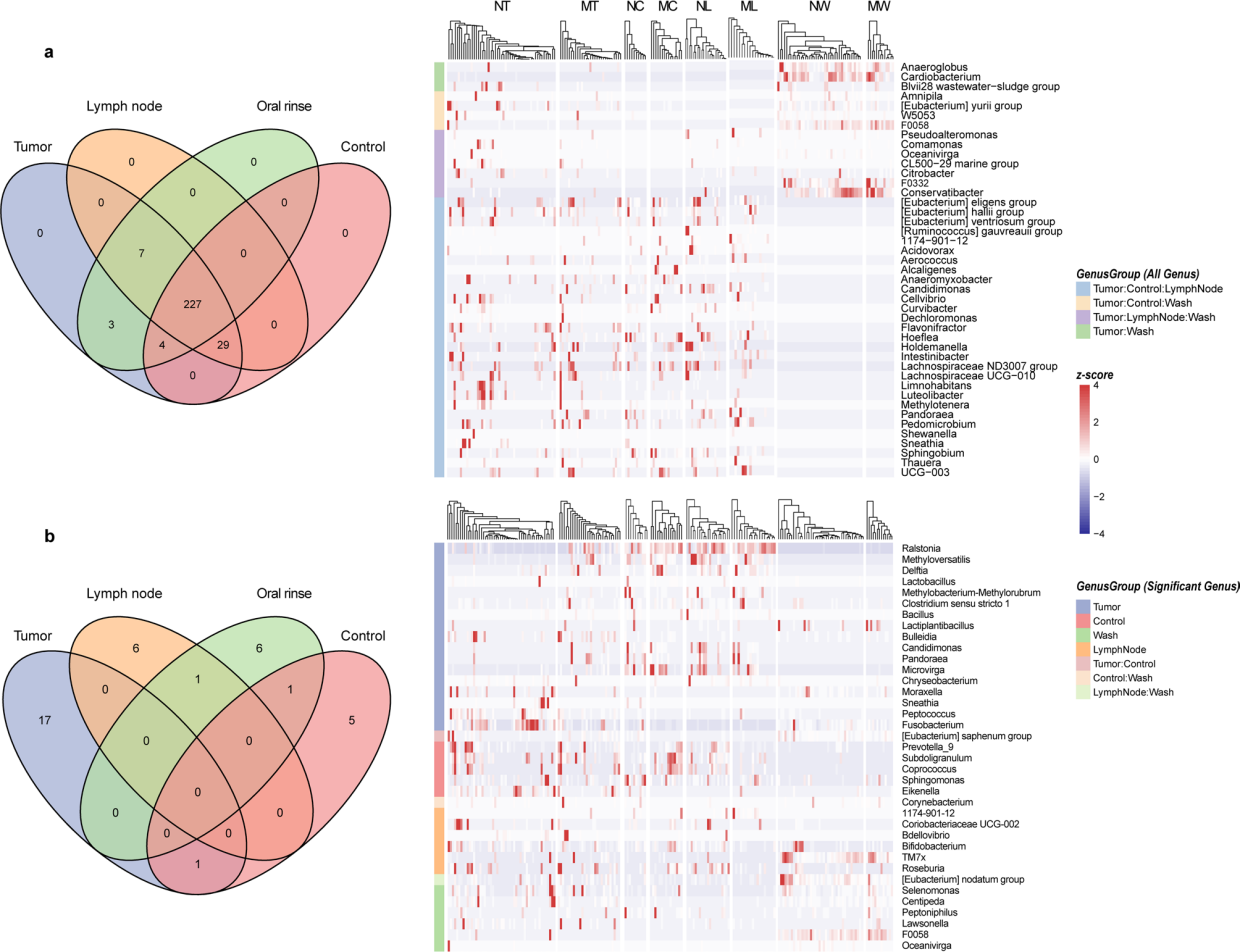
Venn diagrams illustrate the distribution of microbial communities in tumor tissue, adjacent normal tissue, lymph node tissue, and oral rinse samples. **(a)** Overlapping and unique genera across sample types are presented, with a heatmap depicting the relative abundances of genera. **(b)** Overlapping and distinct distributions of differentially abundant genera across sample types are shown, further visualized by a heatmap. NT, tumor tissues from LN- patients; MT, tumor tissues from LN + patients; NC, adjacent normal tissues from LN- patients; MC, adjacent normal tissues from LN + patients; NL, non-metastatic lymph node tissues; ML, metastatic lymph node tissues; NW, oral rinses from LN- patients; MW, oral rinses from LN + patients.

Venn diagram analysis of microbial community distribution revealed shared and unique microbial features across different sample types in LSCC (Fig. 4a). At the genus level, 227 genera were universally present in all four sample types, with no genus exclusively unique to any single sample type. Additionally, 29 genera, including *1174-901-12*,* Pandoraea*,* Hoeflea*, and *Sphingobium*, were shared among tumor tissues, adjacent normal tissues, and lymph node tissues but were absent in oral rinse samples. And 7 genera (*Pseudoalteromonas*,* Comamonas*,* Oceanivirga*,* CL500-29 marine group*,* Citrobacter*,* F0332*, and *Conservatibacter*) were detected in tumor tissues, lymph node tissues, and oral rinse samples, but not in adjacent normal tissue. Notably, all genera identified in lymph node tissues were concurrently observed in tumor tissues.

We then examined the distribution of genera that exhibited significant differences in relative abundance between the LN + and LN- groups across different sample types (Fig. 4b). No genera exhibited significant abundance differences in all four sample types. However, 17 genera (*Ralstonia*,* Bulleidia*,* Fusobacterium*, etc.) showed differential abundance exclusively in tumor tissues between the LN + and LN − groups. Distinctly differentially abundant genera were also identified in adjacent normal tissues, lymph nodes, and oral rinses. Furthermore, *[Eubacterium] nodatum group* was shared between lymph node and oral rinse samples, showing consistent enrichment in LN- groups for both sample types.

### Functional analysis of metastatic and non-metastatic LSCC patients

**Fig. 5:**
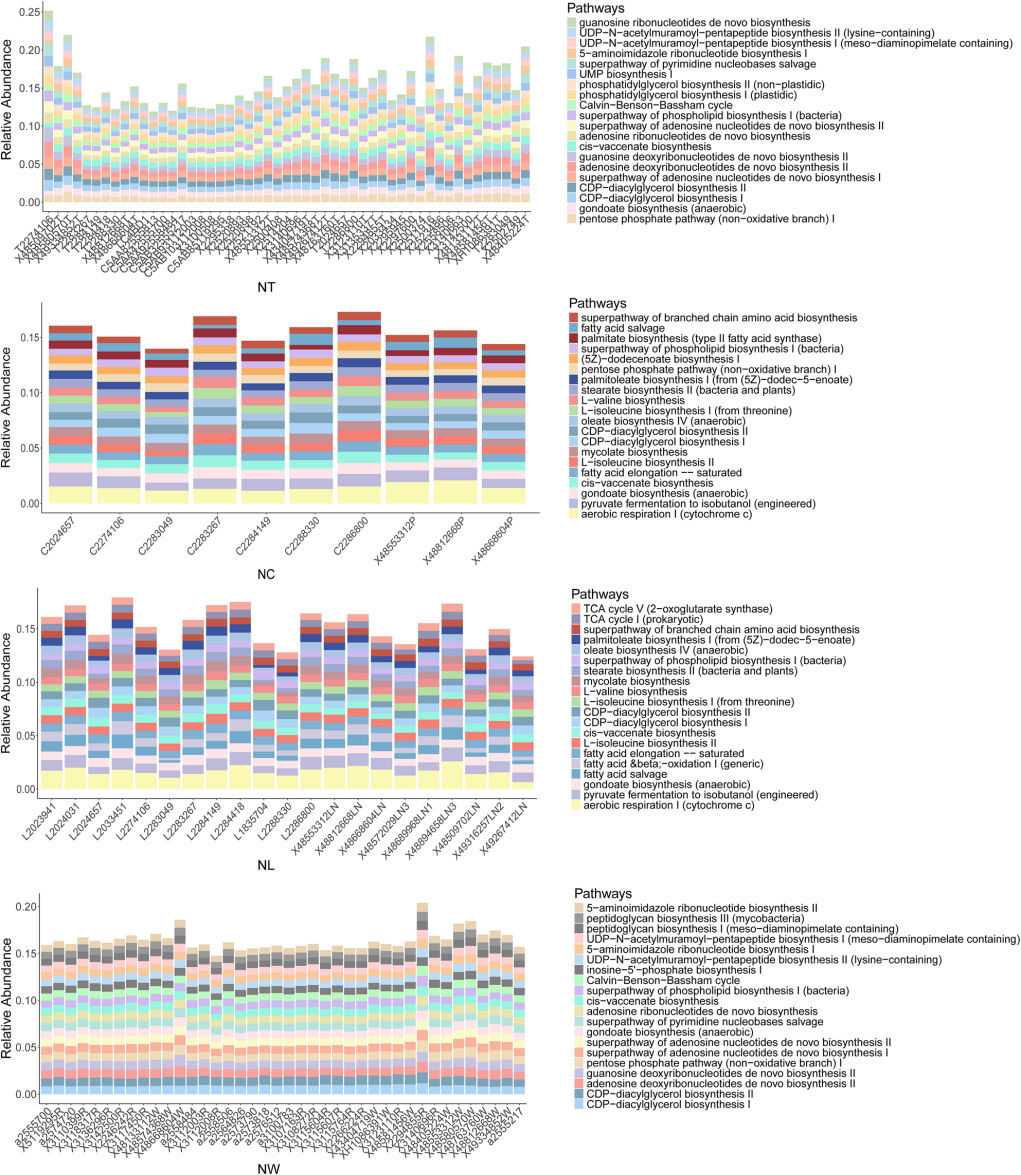

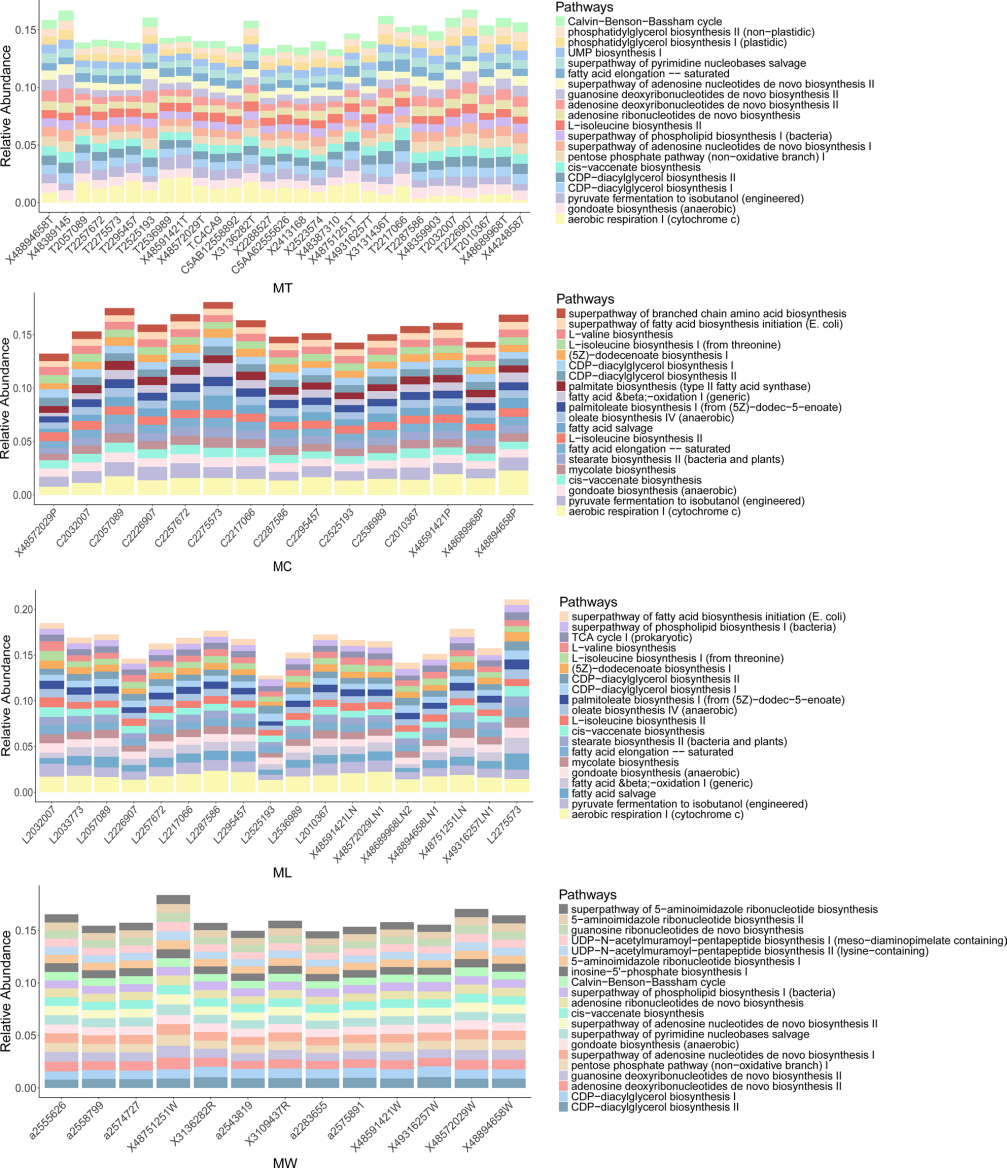
The relative abundance of the top 20 predicted microbial metabolic pathways in tumor tissue, adjacent normal tissue, lymph node (LN) tissue, and oral rinse samples. NT, tumor tissues from LN- patients; MT, tumor tissues from LN + patients; NC, adjacent normal tissues from LN- patients; MC, adjacent normal tissues from LN + patients; NL, non-metastatic lymph node tissues; ML, metastatic lymph node tissues; NW, oral rinses from LN- patients; MW, oral rinses from LN + patients.

The relative abundances of the top 20 predicted metabolic pathways were compared between specimens from the LN + and LN- groups (Fig. 5). Tumor tissues were enriched in pathways related to nucleotide metabolism (e.g., UMP biosynthesis I, superpathway of adenosine nucleotides de novo biosynthesis II), carbohydrate utilization (e.g., Calvin-Benson-Bassham cycle, pentose phosphate pathway [non − oxidative branch] I), and lipid biosynthesis (e.g., phosphatidylglycerol biosynthesis II [non − plastidic], CDP − diacylglycerol biosynthesis I). In contrast, adjacent normal tissues and lymph node tissues exhibited more similar functional profiles, characterized by pathways associated with energy metabolism, fatty acid synthesis, and amino acid biosynthesis, such as aerobic respiration I (cytochrome c), palmitate biosynthesis (type II fatty acid synthase) and superpathway of branched chain amino acid biosynthesis. Oral rinse samples displayed a distinct functional profile, including pathways involving not only nucleotide and carbohydrate metabolism, but also peptidoglycan biosynthesis (peptidoglycan biosynthesis III). In addition, inter-individual variability in pathway distribution within each group was relatively low across all sample types, indicating functional consistency among patients with the same lymph node metastasis status.

**Fig. 6:**
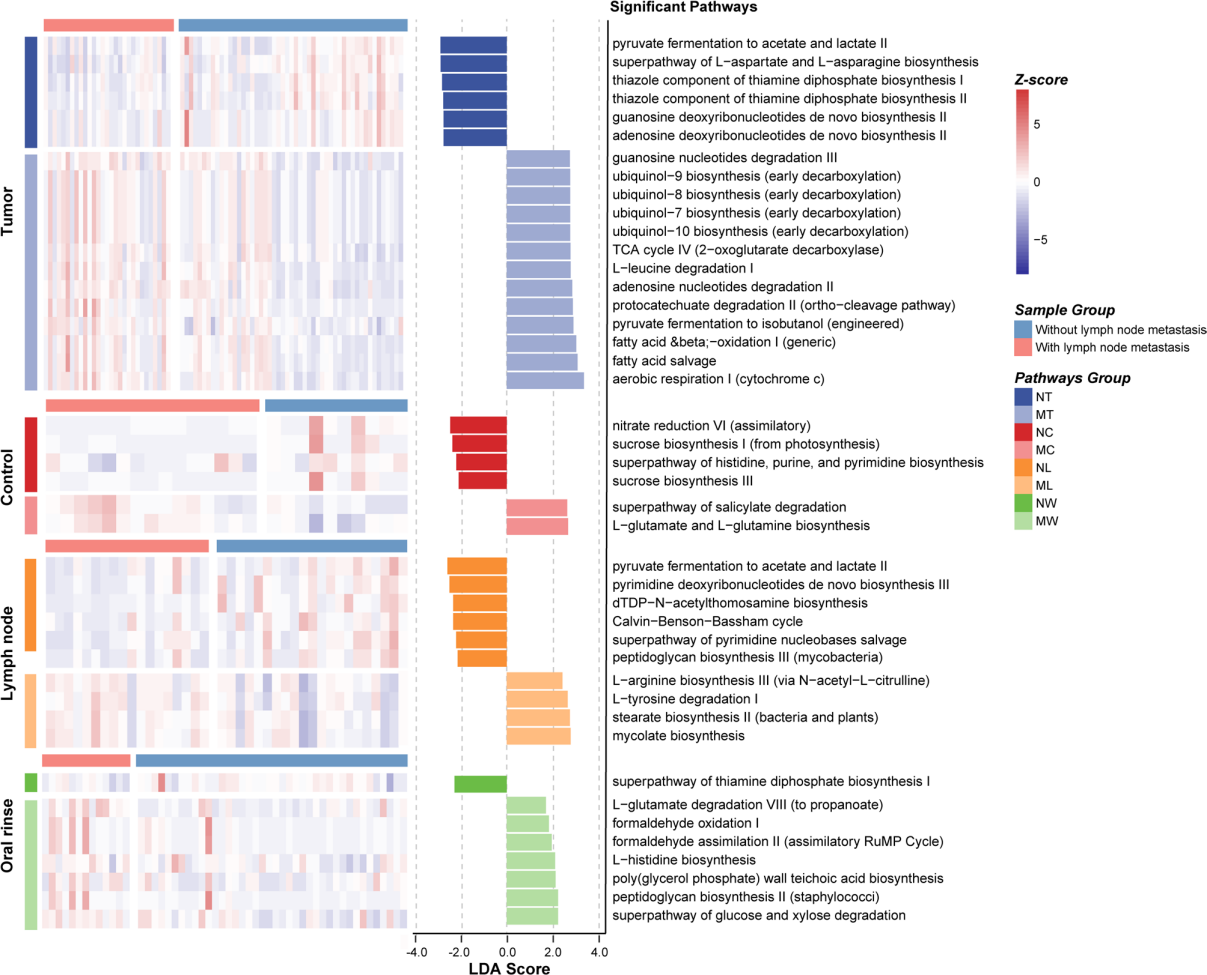
Differential predicted microbial metabolic pathways between LN + and LN- groups in LSCC patients across tumor tissue, adjacent normal tissue, lymph node tissue, and oral rinse samples. A linear discriminant analysis (LDA) score threshold of |LDA Score| ≥ 1.5 was used to identify pathways with significant differences between LN + and LN- LSCC patients across different sample types. The bar plot displays these differentially abundant pathways, while the heatmap illustrates their relative abundance. Only pathways with LDA > 2.5 are shown in tumor tissues due to the large number of differentially abundant functions. NT, tumor tissues from LN- patients; MT, tumor tissues from LN + patients; NC, adjacent normal tissues from LN- patients; MC, adjacent normal tissues from LN + patients; NL, non-metastatic lymph node tissues; ML, metastatic lymph node tissues; NW, oral rinses from LN- patients; MW, oral rinses from LN + patients.

LEfSe analysis further revealed differentially abundant metabolic pathways between LN + and LN- groups across the four sample types (Fig. 6). In tumor tissues, pathways related to aerobic energy production (e.g., aerobic respiration I [cytochrome c]) and ubiquinol biosynthesis were enriched in LN + tumor tissues, while LN- tumors were characterized by pathways associated with basic biosynthetic functions, including pyruvate fermentation to acetate and lactate II and superpathway of L-aspartate and L-asparagine biosynthesis. In metastatic lymph node tissues, mycolate biosynthesis, which is a pathway associated with bacterial pathogenicity, was significantly enriched. And in oral rinse samples, the LN + group showed increased abundance of pathways involved in bacterial cell wall synthesis, such as poly (glycerol phosphate) wall teichoic acid biosynthesis and peptidoglycan biosynthesis II (staphylococci).

### Microbial classification models for stratifying LSCC patients by lymph node metastasis status

**Fig. 7:**
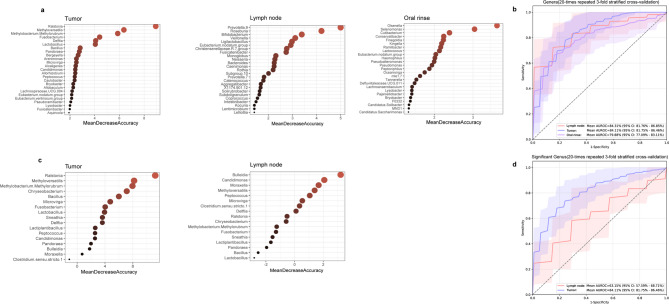
Identification of microbial biomarkers for lymph node metastasis in LSCC patients by random forest models. **(a)** Mean Decrease Accuracy (MDA) values from random forest models based on 25 selected microbial biomarkers in tumor tissues, lymph nodes, and oral rinses. **(b)** Receiver operating characteristic (ROC) curves and area under the curve (AUC) values for tumor tissues, lymph nodes, and oral rinses. **(c)** MDA values of 17 tumor-specific differentially abundant genera in tumor tissues and lymph nodes. **(d)** ROC curves and AUC values based on the 17 tumor-specific differentially abundant genera in tumor tissues and lymph nodes.

To evaluate the potential of microbial features in distinguishing LSCC patients with and without lymph node metastasis, we developed random forest classifiers using microbial genera from tumor tissues, lymph node tissues, and oral rinses. For each sample type, 25 key genera were selected as classification features (Fig. 7a). The classifier based on lymph node tissues achieved an AUC of 84.31% (95% confidence interval [CI]: 81.76% − 86.85%), followed by tumor tissues (AUC = 84.11%, 95% CI: 81.75% − 86.46%) and oral rinses (AUC = 79.88%, 95% CI: 77.09% − 83.11%) (Fig. 7b). To evaluate biological relevance, PERMANOVA analyses were applied to the 25 key genera from each sample type. Significant differences in microbial community structure between LN + and LN- groups were identified in tumor tissues (*P* = 0.0001), lymph node tissues (*P* = 0.0006), and oral rinses (*P* = 0.005) (Supplementary Figure S2).

We further explored the classification potential of 17 tumor-specific differentially abundant genera (*Ralstonia*,* Bulleidia*,* Fusobacterium*, etc.) between the LN + and LN- groups (Fig. 7c). A classifier trained on these 17 genera effectively distinguished LN + from LN- patients in tumor tissues (AUC = 84.11%, 95% CI: 81.75% − 86.46%) but showed moderate performance in lymph node tissues (AUC = 63.15%, 95% CI: 57.59% − 68.71%) (Fig. 7d).

## Discussion

Recent studies have provided preliminary evidence of associations between the microbiome and cervical lymph node metastasis in oral and oropharyngeal cancers^18,19^. However, these studies were limited to either tissue or oral specimens, and research on this association in LSCC remains scarce. In this study, we characterized the microbial profiles of tumor tissues, adjacent normal tissues, lymph node tissues, and oral rinses from LSCC patients with and without lymph node metastasis. Our findings revealed that the microbial features within the primary tumor were significantly associated with cervical lymph node metastasis. Furthermore, the lymph node microbiome substantially overlapped with the tumor microbiome, suggesting potential microbial dissemination between primary tumors and regional lymph nodes. These findings offer new insights into microbiota-associated mechanisms potentially involved in lymph node metastasis of LSCC. Functional predictions further indicated distinct microbial pathways between LN + and LN- groups, including enrichment in lipid biosynthesis, energy metabolism, and cell wall synthesis in LN + patients. Additionally, the classifier based on oral microbiome presented promising potential as a non-invasive approach for detecting occult lymph node metastasis in LSCC. To our knowledge, this is the first study to integrate tumor, adjacent normal tissue, lymph node, and oral microbiome data to investigate lymph node metastasis in LSCC.

We observed significant differences in microbial composition and structure between LN + and LN- primary tumors in LSCC, suggesting a potential association between intratumoral microbial features and lymph node metastasis. Microbial communities were also identified within regional lymph nodes in our study, consistent with previous reports^19,24^. Notably, Venn diagram analysis revealed that all microbial taxa detected in lymph nodes were concurrently present in tumor tissues, while tumor tissues harbored unique taxa absent in lymph nodes, indicating the possibility of a microbiota-associated connection between tumors and lymph node. This hypothesis is supported by existing evidence. Bullman et al. reported that *Fusobacterium nucleatum* strains in primary colorectal tumors and their corresponding liver metastases exhibited over 99.9% genomic similarity, with highly conserved microbial profiles between the two sites^25^. Similarly, a breast cancer mouse model study confirmed shared microbial features between primary breast tumors and lung metastases, distinct from normal breast tissue, which was later validated in human samples^26^.

Physical dissemination via lymphatic or hematogenous routes may represent a potential mechanism by which intratumoral microbiome influences lymph node metastasis^27^. Specifically, intratumoral microbiome could either migrate alongside circulating tumor cells (CTCs) to distant sites^26^ or spontaneously translocate from primary tumors to potential metastatic niches before tumor cell colonization^28^. In our study, the detection of tumor-overlapping microbiome in both LN + and LN- lymph nodes suggests the possibility of microbial dissemination between the tumor and lymph node. Future studies incorporating techniques such as fluorescence in situ hybridization (FISH), immunohistochemistry (IHC), and microbial strain-level genotyping could help clarify whether microbial translocation occurs during lymph node metastasis and, if so, explore the underlying mechanisms.

We also identified sample-specific differentially abundant genera across tumors, adjacent normal tissues, lymph nodes, and oral rinses in both LN + and LN- patients. *Ralstonia* was a tumor-specific differentially abundant genus, with a significantly higher relative abundance in LN + tumor tissues compared to LN- tumors. In gastric cancer studies, *Ralstonia* has been found to be more abundant in gastric mucosa during intestinal metaplasia and early-stage gastric cancer than in superficial gastritis, suggesting its potential role in early carcinogenesis^29–31^. Additionally, *Bifidobacterium* were enriched in non-metastatic lymph nodes compared to metastatic counterparts. Previous studies have shown that *Bifidobacterium* are closely associated with NK cells and can enhance their function within the tumor microenvironment, leading to tumor regression^32^. Based on this, we hypothesize that the enrichment of *Bifidobacterium* in LN- lymph nodes may exert a similar effect, potentially inhibiting tumor cell colonization and growth. Further functional experiments and large-scale cohort studies are needed to validate this hypothesis.

In addition to taxonomic comparisons, we also performed functional profiling of the microbial communities. The results revealed that microbial communities in LN + patients exhibit distinct functional features, with enrichment in pathways related to lipid biosynthesis, energy metabolism, and cell wall synthesis. These differences between LN + and LN- groups were particularly evident in tumor and oral rinse samples and may reflect microbial adaptation to the tumor microenvironment or lymph node metastasis status. The relatively low intra-group variability suggests a high degree of functional consistency within each group. Given that these findings are based on predictive analyses, further validation using metagenomic or metabolomic approaches is warranted.

Using a random forest model, we evaluated the classification power of multi-site microbiota in distinguishing LSCC patients with and without lymph node metastasis. Models based on microbiome from lymph node and tumor tissues demonstrated comparable classification performance, with AUCs of 84.31% (95% CI: 81.76–86.85%) and 84.11% (95% CI: 81.75–86.46%), respectively. Although the oral rinse-based model showed slightly lower efficacy (AUC = 79.88%, 95% CI: 77.09% − 83.11%), its non-invasive nature and clinical accessibility highlight its clinical translational potential, allowing for potential longitudinal monitoring. Additionally, oral rinse samples exhibited lower intra-group variability compared to tissue specimens, consistent with our previous findings^17^. This microbial stability further supports the potential of the oral microbiome as a non-invasive biomarker for detecting occult lymph node metastasis. Although still exploratory, microbiome-based tools may provide additive value for conventional imaging or pathological assessment. Further validation in larger cohorts and standardization of workflows are needed to facilitate clinical translation.

To probe the ecological relevance of the genera selected by the random forest models, we conducted PERMANOVA analyses based on the 25 key genera identified in each sample type (Supplementary Figure S2). The significant separation between LN + and LN- samples across all three sample types (*P* < 0.001) indicates that the genera contributing to model classification also reflect potentially meaningful biological variation associated with metastatic status.

Previous studies have conducted limited comparisons of microbial characteristics across different sample types in head and neck squamous cell carcinoma (HNSCC). For instance, Zeng et al. analyzed saliva, swabs, outer tumor tissues, inner tumor tissues, adjacent normal tissues, and lymph nodes from oral cancer patients^33^. They found that the oral tumor microbiome was primarily clustered by individual, with only minor effects attributable to sample type. And they did not further explore the compositional differences in microbial communities across these sample types. Similarly, Shin et al. compared the microbiota of primary tumors and metastatic lymph nodes in patients with HNSCC, reporting a consistent enrichment of Fusobacterium and a decrease of Streptococcus in both sites compared to healthy tissue^34^. However, this study did not include oral samples. In our study, seven genera were consistently detected in oral, tumor, and lymph node samples but absent in adjacent normal tissues. Four of these shared genera (*Conservatibacter*, *Oceanivirga*, *F0332*, and *Pseudoalteromonas*) were incorporated into the oral microbiota-based classifier. Previous studies have reported an inverse correlation between *Pseudoalteromonas* abundance and breast cancer malignancy^35^ while in lung adenocarcinoma, *Pseudoalteromonas* was significantly enriched in late-stage tumors compared to early-stage patients^36^ indicating its potential role in tumor progression. The biological consistency of these shared genera enhances the reliability of the classifier, and their cross-site persistence suggests a possible “oral-tumor-lymph node” microbial migration pathway involved in metastasis. Although these seven shared genera were not among the 25 key taxa used in tumor and lymph node classifiers, their significance in the oral model suggests that shared microbiome may play a role in early metastatic events, whereas the distinct microbiota of tumors and lymph nodes may reflect dynamic ecological shifts during later metastatic progression. Nevertheless, these findings warrant further investigation and validation in larger, matched sample cohorts from the same patients.

We further constructed a random forest model using 17 tumor-specific microbial genera (*Ralstonia*, *Bulleidia*, *Fusobacterium*, etc.) that exhibited significant associations with lymph node metastasis in LSCC. The model demonstrated strong classification power in primary tumor tissues (AUC = 84.11%, 95% CI: 81.75% − 86.46%), but reduced efficacy when applied to lymph node specimens (AUC = 63.15%, 95% CI: 57.59% − 68.71%). This finding suggests that, as a collective microbial signature, these 17 genera may play a synergistic role in the early stages of lymph node metastasis by altering the tumor microenvironment and facilitating tumor cell dissemination^37^. The reduced classification performance in lymph nodes may reflect the adaptive evolution of the microbiome following metastasis.

While this study provides novel insights into microbiota-related mechanisms underlying LSCC metastasis, several limitations should be acknowledged. First, our findings necessitate validation in larger cohorts, ideally incorporating multiple matched sample types from the same patients. In particular, intra-patient comparisons between metastatic and non-metastatic lymph nodes within LN + patients would help decrease inter-individual variability and strengthen the biological relevance of observed differences. Furthermore, the current analysis lacks an external validation cohort. Future studies should evaluate the discriminative potential of the identified microbial features in independent datasets or prospective clinical cohorts. Second, current compositional data lack functional validation, warranting integrated multi-omics and experimental approaches to delineate microbiome-related metastatic pathways. Additionally, host factors, such as immune profiles and genetic variants, should be incorporated for a more comprehensive understanding of microbial-host interactions during metastasis progression.

## Conclusion

This multi-sample study identified distinct microbial features associated with lymph node metastasis in LSCC. Significant differences in microbial composition and predicted function were observed between LN + and LN- patients across tumor, lymph node, and oral rinse samples. The substantial overlap between tumor and lymph node microbiota suggests a potential microbial connection between the primary tumor and regional lymph nodes. Moreover, the oral microbiome-based classification model showed potential as a non-invasive tool for detecting lymph node metastasis. These preliminary findings provide a foundation for future studies exploring microbiome-related mechanisms and their clinical applications in LSCC.

## Supplementary Information

Below is the link to the electronic supplementary material.

Supplementary Material 1

Supplementary Material 2

Supplementary Material 3

Supplementary Material 4

Supplementary Material 5

## Data Availability

The datasets generated and/or analysed during the current study are available in the NCBI (SRA) repository, available at https://www.ncbi.nlm.nih.gov/bioproject/PRJNA1256458. The accession number is PRJNA1256458. Supplementary Table S4 provides a detailed mapping of Patient IDs, Sample IDs, SRA sample names, and Sample types (tumor, adjacent normal tissue, lymph node, and oral rinse) to facilitate cross-referencing and ensure data traceability.
