# Supplementary material for: Microbiome characteristics associated with lymph node metastasis in laryngeal squamous cell carcinoma
Source: Sci Rep. 2025 Aug 24;15:31123. doi: 10.1038/s41598-025-16431-5 (PMC12375765; doi:10.1038/s41598-025-16431-5)
Supplement: Supplementary file 1 — Supplementary Material 1 [file 41598_2025_16431_MOESM1_ESM.pdf]

Microbiome characteristics associated with lymph node metastasis in laryngeal squamous cell carcinoma

Fangxu Yan[#], Shibo Chen[#], Xin Xia, Yue Fan, Shuting Yu, Xiao Zhang and Xingming Chen

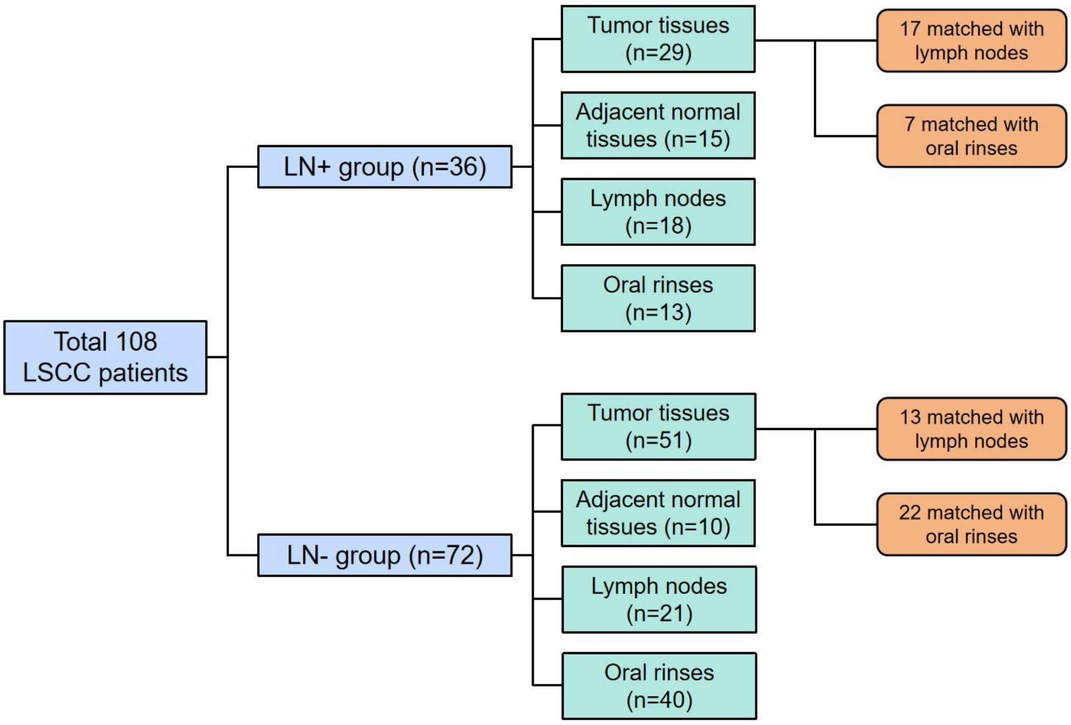

Supplementary Figure S1 Flowchart illustrating the distribution and matching status of collected samples from 108 LSCC patients. LN, lymph node; LSCC, laryngeal squamous cell carcinoma.

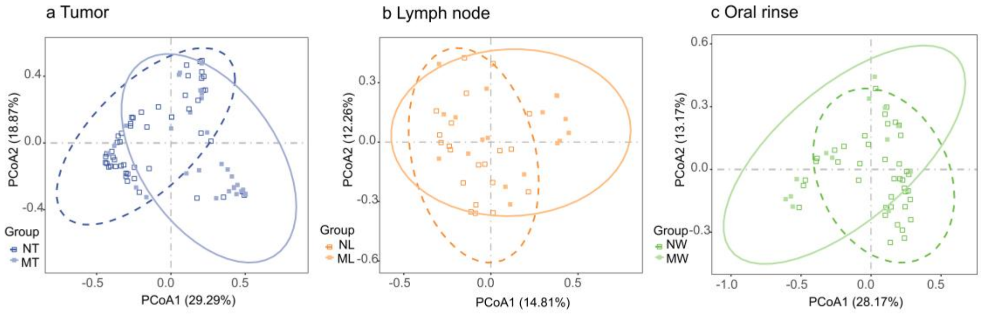

d

Group1	Group2	PERMANOVA.R2	PERMANOVA.pvalue
NT	MT	0.077301033	0.0001
NL	ML	0.053739932	0.0006
NW	MW	0.056763091	0.0050

Supplementary Figure S2 Principal coordinate analysis (PCoA) based on Bray-Curtis distances using the top 25 genera identified by the random forest model. Plots show microbial community structure differences between LN+ and LN- groups in (a) tumor tissues, (b) lymph node tissues, and (c) oral rinse samples. PERMANOVA P-values indicate statistically significant separation between groups in all three sample types. LN, lymph node; NT, tumor tissues from LN- patients; MT, tumor tissues from LN+ patients; NC, adjacent normal tissues from LN- patients; MC, adjacent normal tissues from LN+ patients; NL, non-metastatic lymph node tissues; ML, metastatic lymph node tissues; NW, oral rinses from LN- patients; MW, oral rinses from LN+ patients; PERMANOVA, permutational multivariate analysis of variance.
